# Patterns of immune evasion in triple-negative breast cancer and new potential therapeutic targets: a review

**DOI:** 10.3389/fimmu.2024.1513421

**Published:** 2024-12-13

**Authors:** Lucía Serrano García, Beatriz Jávega, Antonio Llombart Cussac, María Gión, José Manuel Pérez-García, Javier Cortés, María Leonor Fernández-Murga

**Affiliations:** ^1^ Medical Oncology Department, Hospital Arnau de Vilanova, Fundación para el Fomento de la Investigación Sanitaria y Biomédica de la Comunitat Valenciana (FISABIO), Valencia, Spain; ^2^ Grupo Oncología Traslacional, Facultad de Ciencias de la Salud, Universidad Cardenal Herrera-Centro de Estudios Universitarios (CEU), Alfara del Patriarca, Spain; ^3^ Medica Scientia Innovation Research (MEDSIR), Oncoclínicas & Co., Jersey City, NJ, United States; ^4^ Medical Oncology Department, Hospital Ramon y Cajal, Madrid, Spain; ^5^ International Breast Cancer Center (IBCC), Pangaea Oncology, Quiron Group, Barcelona, Spain; ^6^ Universidad Europea de Madrid, Faculty of Biomedical and Health Sciences, Department of Medicine, Madrid, Spain

**Keywords:** triple negative breast cancer, immunosuppression, immunotherapy, therapeutic target, signaling pathway, tumor microenvironment

## Abstract

Triple-negative breast cancer (TNBC) is an aggressive subtype of breast cancer characterized by the absence of progesterone and estrogen receptors and low (or absent) HER2 expression. TNBC accounts for 15-20% of all breast cancers. It is associated with younger age, a higher mutational burden, and an increased risk of recurrence and mortality. Standard treatment for TNBC primarily relies on cytotoxic agents, such as taxanes, anthracyclines, and platinum compounds for both early and advanced stages of the disease. Several targeted therapies, including bevacizumab and sunitinib, have failed to demonstrate significant clinical benefit in TNBC. The emergence of immune checkpoint inhibitors (ICI) has revolutionized cancer treatment. By stimulating the immune system, ICIs induce a durable anti-tumor response across various solid tumors. TNBC is a particularly promising target for treatment with ICIs due to the higher levels of tumor-infiltrating lymphocytes (TIL), increased PD-L1 expression, and higher mutational burden, which generates tumor-specific neoantigens that activate immune cells. ICIs administered as monotherapy in advanced TNBC yields only a modest response; however, response rates significantly improve when ICIs are combined with cytotoxic agents, particularly in tumors expressing PD-L1. Pembrolizumab is approved for use in both early and advanced TNBC in combination with standard chemotherapy. However, more research is needed to identify more potent biomarkers, and to better elucidate the synergism of ICIs with other targeted agents. In this review, we explore the challenges of immunotherapy in TNBC, examining the mechanisms of tumor progression mediated by immune cells within the tumor microenvironment, and the signaling pathways involved in both primary and acquired resistance. Finally, we provide a comprehensive overview of ongoing clinical trials underway to investigate novel immune-targeted therapies for TNBC.

## 1 Introduction

### 1.1 Epidemiologic and clinical features of triple-negative breast cancer

Triple-negative breast cancer (TNBC) is characterized by the absence of estrogen and progesterone receptors and the absence of over-expression of the human epidermal growth factor 2 (HER-2). TNBC is a highly aggressive subtype, accounting for 15-20% of all breast cancers (1). TNBC expresses cytokeratins 5, 14 and 17 and it usually present as an undifferentiated histological grade with high Ki-67 expression, with a high proliferative capacity (2). The mean age at diagnosis is approximately 50 years, which is the lowest of all breast cancer (BC) subtypes. The most aggressive TNBC tumors are associated with younger age at diagnosis (3). The aggressiveness of these tumors is mainly due to their invasiveness, metastatic capacity, and treatment resistance. While specific treatments are available for other types of breast cancer, such as hormonal therapy for luminal cancers and HER-2 blockers for HER-2 positive cancers, TNBC lacks therapeutic targets. Consequently, the only treatment options are conventional therapies such as surgery, chemotherapy (taxanes, platinum, and anthracyclines), and radiotherapy. In TNBC, the risk of distant recurrence is high and progression-free survival (PFS) and overall survival (OS) are poor. Five-year OS in early TNBC is only 77% vs. 91% in all BCs. In metastatic TNBC, the 5-year OS is only 12% (4–6).

### 1.2 Immunoediting in TNBC

The tumor microenvironment (TME) in most breast cancer subtypes is composed not only of cancer cells, but also of other cellular components such as immune cells, fibroblasts, adipocytes, and other elements such as extracellular matrix and soluble factors. The TME in TNBC—the most invasive subtype—is unique and differs from other subtypes in several ways. TNBC tumors develop distinct signaling pathways that reprogram the environmental cells to promote tumor progression. In turn, this forms a positive feedback loop that can affect tumor cell-targeted therapy (7). Consequently, it is important to study the unique characteristics of TNBC in order to improve diagnosis and provide more effective treatments.

It is essential to understand how the tumor interacts with the immune system and the TME in order to determine the mechanisms underlying cancer cell growth, invasion, and metastasis. Under pathological conditions, tumor cell-induced leukocyte inactivation contributes to tumor progression (8). In recent decades, intensive research into the process of carcinogenesis and the role of the immune system has led to the cancer immunoediting hypothesis, which postulates three phases in oncogenesis: elimination, equilibrium, and escape (9). Some tumor cells arise in the equilibrium or escape phases, without entering the elimination phase. External factors can also modify the flow of the phases (10).

In the first phase of cancer immunoediting—the elimination phase—the innate and adaptive immune systems act in coordination to eliminate tumor cells. However, if the tumor cells manage to survive this initial phase, the cancer can progress to the equilibrium phase in which there is a balance between malignant cells and the immune system. In this phase, the cancer cells are unable to evade immune surveillance but the immune system cannot eliminate the cancer cells (11). In the escape phase, the immune system loses its ability to control or restrict the growth of the tumor cells. The complex interaction between stromal cells and cancer cells forms a complex immunosuppressive microenvironment, which triggers an imbalance between the immune system and the tumor (9).

In this review, we evaluate the factors—primarily the TME and alterations in signaling and metabolic pathways—that can promote immune escape and lead to immune evasion and treatment resistance. We also review recent strategies aimed at reversing these alterations. Finally, we discuss the current landscape for immunotherapy in TNBC.

## 2 Myeloid cells in the tumor microenviroment

**Figure 1:**
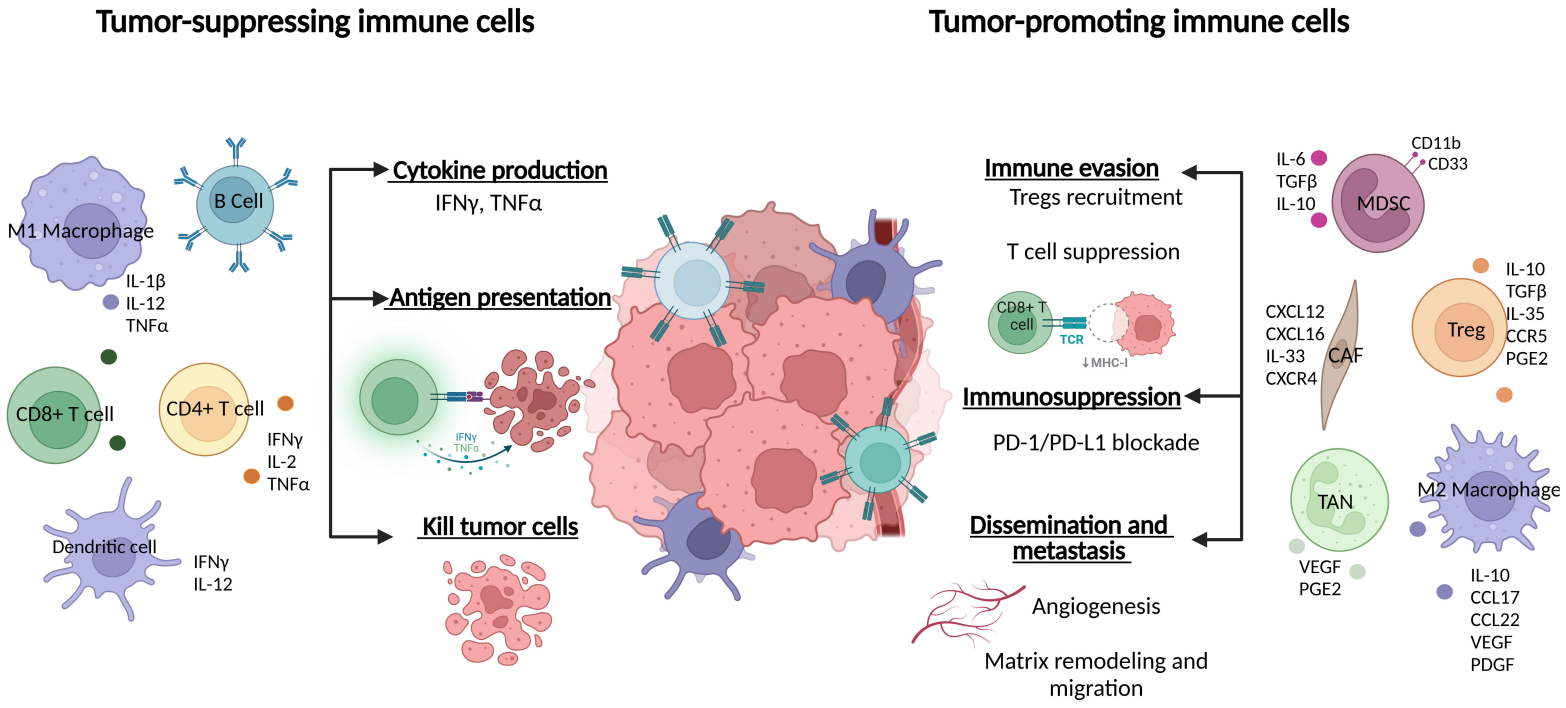
Composition of the tumor microenvironment. TME, tumor microenvironment; TAM, tumor-associated macrophages; DC, dendritic cells; MDSC, myeloid-derived suppressor cells; Treg, regulatory T cell; TAN, tumor-associated neutrophils. CAF, Cancer-associated fibroblasts; IL, Interleukin; TNF, Tumor Necrosis Factor; IFN, Interferon; VEGF, Vascular Endothelial Growth Factor; TGF, Transforming growth factor; CXCL, chemokine (C-X- C motif) ligand; CCL, chemokine (C-C motif) ligand; PGE2, Prostaglandin E2.

Immature myeloid cells (iMC) are cells involved in the innate immune response against pathogens. These cells play a critical role in tissue repair after pathogen elimination (12). Myeloid cells are important at all stages of tumor progression and drive both the innate and adaptive immune response (13–15) (**Figure 1**). Early in tumor development, iMCs—including macrophages and dendritic cells (DC)—trigger an inflammatory response to induce myelopoiesis and recruit other immune system cells to help eliminate cancer cells (11, 13, 16–18). However, if the iMCs are unable to eliminate the tumor cells, the result is persistent inflammation, which leads to the continuous recruitment of more immune cells that may be reprogrammed to promote tumor development (19–22). Immature MCs include the macrophages, DCs, neutrophils, monocytes, and myeloid-derived suppressor cells (MDSC) that characterize the TME. These cells have distinct but overlapping functions (**Table 1**). In the next section, we discuss the mechanisms of MC-mediated immunity and tumor evasion in TNBC.

**Table 1:** Summary of the cells involved in tumor immunosuppression and their effects in TNBC.

Immunosuppressive cell	Phenotype	Product	Pro-tumor mechanism	Effect in TNBC	Ref (s)
Tumor-associated macrophages	CD68^+^ CD163^+^	IL-10, CCL17, CCL22	Inhibition of T cell recruitment and activity, recruitment of Tregs	Immune evasion and ICIs resistance	(23, 24)
		VEGF, PDGF	Angiogenesis	Tumor maintenance and metastasis	(25)
		MMP proteins family	Matrix remodeling and migration	Dissemination and metastasis	(25)
Tumor associated neutrophils	CD14^−^CD15^+^ CD66b^+^CD16^+^	MMP9	Extracellular matrix degradation, EMT, intravasation	Tumor formation and metastasis	(26)
		VEGF	Angiogenesis	Dissemination and metastasis	(26)
		Mitochondrial DNA fibers and proteases	NETs formation, invasion, and migration	Lung metastases	(27)
		PGE2	NK inhibition	Immune evasion and tumor progression	(28)
		Arg1 and iNOS	CD8+ T cell inhibition	Immune evasion and tumor progression	(29)
Myeloid-derived suppressor cells	HLA-DR^-^, CD33^+^, CD11b^+^, CD14^+/-^, CD15^+/-^	ROS and RNS	TCRs inactivation	Immune evasion	(30–32)
		Arginase	Cytotoxic T cell depletion	Immune evasion	(33, 34)
		IDO	T cell suppression and Tregs recruitment	Immune evasion	(35)
		TGF-β and IL-10	Inhibition of proinflammatory cytokines produced by Macrophages and T cells	Immune evasion	(36)
Cancer associated fibroblasts	CD34^+^PDGFRβ^+^PDGFRα^+^ CD146^-^	SPARC and MMP proteins	Inhibition of TNBC cell adhesion	Invasion and metastasis	(37, 38)
		Biglycan	Reduction of CD8+ T cells	Immune evasion	(39)
		CXCL12	Treg maturation	Immune evasion	(40, 41)
		CD73 and	Treg infiltration	Immune evasion	(42)
		CXCL16	M-MDSCs recruitment	Immune evasion	(43)
		IL-33	Type 2 immunity promotion	Immune evasion and lung metastasis	(44)
		CXCL12-CXCR4 axis	Monocyte differentiation into LAMs and T cell inhibition	Immune evasion	(45)
		Endo180 receptor	Recruitment of Tregs and MDSCs, and T cell exclusion	Immune evasion and ICI resistance	(46)
T regulatory lymphocytes (Tregs)	CD4^+^CD25^+^ Foxp3^+^	IL-10, TGFβ, IL-35 and PGE2	Inhibition of DC and T cell function. Promotion of MDSCs function.	Immune evasion	(47, 48)
		CCR5 and associated chemokines	Cell recruitment and inflammation	Lymph node invasion Metastasis	(47)
		Granzyme and perforin	Death of T cells and NK cells	Immune evasion	(48)

ICIs, Immune checkpoints Inhibitors; VEGF, Vascular Endothelial Growth Factor; PDGF, Platelet Derived Growth Factor; MMP, Matrix metalloproteinase, EMT, Epithelial to Mesenchymal Transition, NETs, Neutrophil Extracellular Tramps; PGE2, Prostaglandin E2; NK, Natural Killer, Arg, Arginase, iNOS, inducible Nitric Oxide Synthase, ROS, Reactive Oxygen Species, RNS, Reactive Nitrogen Species, IDO, Indoleamine Dioxygenase, TGF-β, Transforming Growth Factor β, IL, Interleukin; CCL, chemokine (C-C motif) ligand; SPARC, Secreted Protein Acidic And Cysteine Rich; PDGFR, Platelet Derived Growth Factor Receptor, CXCL, chemokine (C-X- C motif) ligand; CCR, chemokine (C-C motif) Receptor; LAMs, Lipid Associated Macrophages.

### 2.1 Tumor-associated macrophages

Tumor-associated macrophages (TAM) are a class of immune cells located in the TME. These cells play a significant role in cancer cell survival and progression (49–51). In breast cancer, TAMs are abundant in the TME. This is especially true in TNBC, where TAMs may constitute more than 50% of cells in the TME (52). These macrophages are involved in the development of TNBC, including the initial onset, through metastasis. The percentage of TAMs in the TME is directly associated with the risk of metastasis and poor survival (53). Cytokines from the TME can induce phenotypic changes in macrophages through a process called TAM polarization. Depending on the signal stimulation of the TME, TAMs can polarize into two distinct phenotypes: the classically activated macrophages (M1) under proinflammatory conditions (IFNγ, TNFα, GM-CSF) or the alternatively activated (M2) macrophages through IL-10, TGFβ, IL-4 and IL-13 (54). In general, M1-TAMs are associated with less invasiveness, whereas M2-TAMs are associated with greater invasiveness, tumor growth, and poor prognosis (55). M2-TAMs are the predominant phenotype in BC (56).

M1-TAMs, characterized by CD68 expression, highly express inducible nitric oxide synthase and TNF-α. They exert their anti-tumor activity by promoting pro-inflammatory and immune responses. By contrast, M2-TAMs are characterized by CD68 and CD163 expression, secretion of IL-10, CCL17 and CCL22, and elevated expression of mannose, scavenger, and galactose receptors (25). M2-TAMs are involved in the stimulation of tumor angiogenesis, matrix remodeling, and tumor cell migration and invasion. They play an important role in immune suppression and tissue repair (23, 57, 58).

Recent research shows that anti-programmed cell death ligand (PD-L1) antibodies can potentially inhibit M2 polarization both *in vitro* and *in vivo*, which may contribute to the anti-metastatic and anti-angiogenic activity of antiPD-L1 therapy in TNBC (59). M2-TAMs suppress the anti-tumor effects of PD-1/PD-L1 blockade through multiple mechanisms, including the hyperactivation of regulatory T cells (Treg) and PD-1/PD-L1 axis modulation. As a regulatory factor that limits the killing effect of T cells, TAMs suppress T-cell immunity (24). Macrophages present in mammary gland tissue are an important source of TAMs, helping to mediate local recurrence and distant metastasis in TNBC (60).

Several studies have described the mutual interaction between TAMs and TNBC cells. TNBC cells have a strong ability to induce M2 macrophage differentiation, which helps the cells to acquire resistance to immunotherapy and chemotherapy, as well as to bromodomain and extra terminal domain (BET) inhibitors (61–63). Co-culture of cancer cells with macrophages greatly enhances cancer cell migration, macrophage infiltration, tumor growth, and cancer metastasis (64).

### 2.2 Tumor-associated neutrophils

Neutrophils are the most common cells in the innate immune system. The normal role of these cells is to eliminate pathogens or tumor cells through the production—during inflammation—of cytokines, proteases, chemokines, reactive oxygen species (ROS), and myeloperoxidase (MPO). Infiltration of MPO-positive neutrophils is a prognostic factor for better OS in BC (65). This phenotype, known as N1, has anti-tumor activity.

Although neutrophils have an anti-cancer role, they can also act as immune suppressor cells (N2 phenotype). In this case, they are known as tumor-associated neutrophils (TAN), which are present in peripheral blood (circulating neutrophils) and in the TME (tumor infiltrating neutrophils) (66). These cells are present in all breast cancers, but TNBC has been shown to present the highest percentage of TANs (67). A higher neutrophil-to-lymphocyte ratio has been associated with worse treatment response and more adverse effects (e.g., anaphylaxis), and decreased PFS and OS in certain cancers, including TNBC (68–70).

Circulating neutrophils can associate with circulating tumor cells (CTC) to form clusters, thereby inducing expression of cell-cycle progression and the proliferation of genes such as Ki-67 (71). Tumor-infiltrating neutrophils secrete proteases such as metalloproteinase 9 (MMP-9), which is responsible for extracellular matrix degradation and activation of vascular endothelial growth factor (VEGF), which promotes epithelial-to-mesenchymal transition (EMT), intravasation, and angiogenesis—all of which stimulate tumor formation and metastasis (26). Metastasis can be further promoted by neutrophil extracellular traps (NET), which are web-like structures mainly made of mitochondrial DNA fibers and proteases that stimulate cancer cell migration and invasion. In BC, aged neutrophils form NETs to promote lung metastasis (27). Park et al. showed that these metastatic processes could be blocked in TNBC by inhibiting the formation of NETs or by degrading them with deoxyribonuclease I (72).

Neutrophils found in lung metastases from primary breast tumors have an immunosuppressive role and are capable of inhibiting natural killer (NK) cells and lung infiltrating lymphocytes mediated by prostaglandin E2 (PGE) (28). In turn, TNBC cells can promote the migration and recruitment of neutrophils via TGF-β and CXCR2 ligand secretion. In response to TGF-β, neutrophils activate arginase 1 (Arg-1) and inducible nitric oxide synthetase (iNOS). These enzymes block lymphocyte T CD8+ recruitment and activity (29). In murine models of BC, proinflammatory cytokines generated by tumor cells, such as interleukin 1 beta (IL-1β), which promotes the expression of IL-17 in γδ T cells, can trigger the polarization of neutrophils towards an N2 phenotype, and expansion of these neutrophils through granulocyte colony-stimulating factor (G-CSF) (73).

### 2.3 Myeloid-derived suppressor cells

Myeloid-derived suppressor cells are a set of innate immature myeloid cells generated by abnormal myelopoiesis in the tumor. Three subsets of MDSCs have been described based on their surface markers, as follows: 1) monocytic-MDSCs (M-MDSC) with a HLA-DR^-/lo^, CD33^+^, CD11b^+^, CD14^+^, CD15^-^ phenotype; 2) granulocytic or polymorphonuclear MDSCs (G-MDSCs or PMN-MDSCs), characterized by HLA-DR^-/lo^, CD33^+^, CD11b^+^, CD14^-^, CD15^+^ phenotype; and 3) early-MDSCs (e-MDSCs), which are defined by the lack of CD14 and CD15 markers (74). MDSCs are one of the most potent immunosuppressor cell groups observed in the TME in several different type of cancers, including breast cancer. The presence of MDSCs has been linked to poor outcomes in TNBC. For example, Elbasateeny et al. found high levels of circulating M-MDSCs in the patients with the worst prognosis; by contrast, patients who obtained a pathological complete response to neoadjuvant chemotherapy (NAC) had a lower percentage of G-MDSCs compared to partial responders (75). Inactivation of MDSCs can restore antitumor immunity, thus allowing the immune system to return to the elimination phase. High expression of the genes responsible for the recruitment of MDSCs and Tregs (CXCL10, CYBB and NCF2) has been correlated with immunosuppression and worse prognosis (76, 77).

Various mechanisms may be involved in immunosuppression by MDSCs. The first of these to be described was the production of free radicals, ROS, and reactive nitrogen species (RNS), which are involved in the generation of peroxynitrite-free radicals due to the interaction with nitric oxide (NO) produced by inducible iNOS, which is highly activated in MDSCs. These species can alter T lymphocyte receptors, thus inhibiting tumor recognition. ROS promotes the recruitment and function of MDSCs (30). ROS also promotes expression of immune checkpoints such as PD-L1 on the surface of MDSCs, which also contributes to T cell dysfunction. Another well-studied mechanism is arginase release, which results in the hydrolyzation of L-arginine, an amino acid required for T cell function and surveillance. The hydrolysis process generates metabolites with immunosuppressive properties (e.g., polyamines), potentially leading to cell proliferation (33). Indoleamine 2,3-dioxygenase (IDO) is an enzyme responsible for tryptophan hydrolysis. IDO is expressed by tumor cells and by MDSCs and has been associated with T cell suppression and recruitment of Tregs. IDO expression in MDSCs has been associated with lymph node metastasis in TNBC (35). Cell culture models of TNBC show that MDSCs increase IDO expression in TNBC and the enzyme itself also promotes MDSC proliferation (78).

MDSCs are strongly implicated in the metastatic process since they promote postoperative recurrence and premetastatic niche formation (79). In a mouse model of TNBC, expansion of MDSCs in hypoxia led to the depletion of CD4+ and CD8+ T cells and lung metastasis (80). Cancer stem cells (CSC) secrete cytokines and chemokines such as G-CSF, granulocyte-macrophage colony-stimulating factor (GM-CSF), CXCL2, and CCL22 due to the ΔNp63 transcription factor, which promotes the development and recruitment of MDSCs to the primary tumor and premetastatic niche (81). In turn, MDSCs attract other immune suppressor cells such as cancer-associated fibroblasts (CAF), and they also produce MMP9 and chitinase 3-like 1 involved in angiogenesis, EMT, and CSC (82). In both melanoma and BC, CTCs form clusters with G-MDSCs to promote the production of ROS and, thereby, immunosuppression. In turn, G-MDSCs potentiate the expansion and metastatic properties of CTCs (83).

MDSCs have been linked to worse treatment response to chemotherapy and immune checkpoint blockers (ICB) (78). That study showed that TNBC tumors with poor response to NAC expressed low levels of circulating tryptophan, which was, in turn, associated with high levels of IDO, e-MDSC, and tryptophan-derived metabolites. This finding suggests that e-MDSCs (induced by and expressors of IDO) could be used as a predictive biomarker of the efficacy of NAC (78). Another study (36) showed that atovaquone, an antiprotozoal drug and MDSC inhibitor, reduced tumor growth in paclitaxel-resistant models of breast cancer (including TNBC), decreasing the production of TGFβ and IL-10 (immunosuppressive cytokines secreted by MDSCs), with a decrease in Tregs at the tumor site (36). Franklin et al. found that chemotherapy-resistant TNBC with mutated KRAS presents elevated levels of the cytokines CXCL1 and CXCL2—chemoattractant for MDSCs (specifically G-MDSCs)—which have been associated with a decrease in several cytokines (CXCL 9, 10, and 11) that promote T lymphocyte recruitment (84). In this same line, another study found that triple-negative tumors enriched in G-MDSCs also had lower response rates to ICB (85).

Given the role of MDSCs described above, these cells could have diverse applications, notably as a promising potential prognostic biomarker and predictor of treatment response. MDSCs may even represent a therapeutic target in TNBC.

### 2.4 Dendritic cells

Dendritic cells (DC) are a major group of antigen-presenting cells (APC) that play an essential role in cancer immunity. DCs are considered central components of the TME. Wang et al. described how these cells recognize and process pathogenic antigens into small peptides and present them to T cells to generate an immune response, thereby acting as a link between innate and adaptive immunity. In addition to transporting tumor antigens to lymph nodes, thus initiating tumor-specific immunity, DCs also maintain antitumor functions by reactivating T cells in the TME (86). DCs are generally divided into conventional DCs (cDC) and plasmacytoid DCs (pDC). Conventional DCs include cDc1 and cDc2 (intracellular antigen presentation through major histocompatibility complex I [MHC-I]/CD8+ T cell and MHCII/CD4+ T cells, respectively) while pDCs are associated with antitumor and antiviral response via the production of interferon (IFN)-γ (87).

Dendritic cells are associated with better outcomes in BC patients (88). Lee et al. demonstrated a positive correlation between the expression of CD11c DCs and TILs in TBNC (89). Wu and colleagues found that CCL19 could be used as a biomarker in TNBC due to the association between mature CCL19+ DCs and response to anti-PD-L1 immunotherapy (90).

Oshi et al. (91) found that genes involved in inflammatory and immune responses (e.g., cytolytic activity and IFN-γ signaling) were highly expressed in patients with high levels of pDCs. Furthermore, high levels of pDCs—but not cDCs— was a strong predictive factor of better disease-free survival (DFS) and disease-specific survival outcomes in patients with TNBC, which suggests that pDC levels may be clinically significant in this patient population (91). By contrast, Michea et al. found that a high z-score (i.e., the distance between a given datapoint and the mean) for pDC was significantly associated with better DFS for luminal BC but not TNBC (92). Given these contradictory findings, more research is needed to clarify the value of pDC levels in TNBC.

Multiple studies have demonstrated that DC-based therapies could potentially offer a new treatment approach for TNBC. In these patients, the DC fusion vaccine could act as a tumor cell antigen presenter to generate a response that, in turn, triggers a specific immune response against malignant cells (93, 94). Mejías et al. recently found that the combination of a DC vaccine with NAC stimulates the antitumor immune system, as evidenced by a post-treatment increase in CD8 levels in TNBC samples (95).

## 3 Tumor infiltrating lymphocytes

Tumor infiltrating lymphocytes are immune cells, mainly T cells but also NK and B cells (in smaller amounts), that play a significant role in the TME. TILs are involved in tumor cell identification and elimination. Crucially, TILs have played an important part in the advancement of immunotherapy (96). The presence of TILs at diagnosis is associated with a better prognosis in TNBC (97). TILs have also been shown to be valuable prognostic and predictive biomarkers in BC (98) (**Figure 1**). In this review, we analyze two subtypes of T cells—Tregs and exhausted T cells—and describe the mechanisms that promote immune escape of tumor cells in TBNC.

### 3.1 Regulatory T lymphocytes

Tumor infiltrating lymphocytes play an essential role in the TME and also limit BC growth and progression. The role of Tregs is of particular interest because these cells are more abundant in TNBC than in other BC subtypes (99). Tumor-infiltrating Tregs interact with tumor and stromal cells and with extracellular matrix components in the TME; they can directly contribute to tumor progression and induce an immunosuppressive phenotype (100). These immune cells are major contributors to the pro-tumor immune response in breast cancer and associated with poor prognosis (10).

Tregs are a specialized subpopulation of T cells, characterized by FOXP3 expression, that act as key mediators of immune tolerance (101). Tregs prevent autoimmunity by inhibiting T cell proliferation and cytokine production. In TNBC, Tregs induce an immunosuppressive microenvironment, inhibit CD8+ and CD4+ T-cell activation, and also inhibit the anti-tumor immune response (47, 102).

Several studies have shown that elevated Treg levels may be a potential biomarker of BC. The presence of high numbers of tumor-infiltrating Tregs has been associated with worse prognosis due to their role in suppressing immune cell activity (103–105). However, other studies have reported contradictory data, showing that a high intratumoral Treg density represents a good prognostic panel in TNBC (106, 107). These inconsistent findings may be due to the method used to identify the Treg cells, as evidenced by a study that found FOXP3+/CD25+ Tregs—but not FOXP3+ Tregs—were positively associated with improved survival in TNBC (108).

Jamiyan et al. assessed expression of stromal FOXP3 Tregs in 107 TNBC samples, finding an association between low levels of stromal FOXP3 Tregs and good prognosis (109). Miyashita et al. evaluated variations in CD8+ T lymphocytes, FOXP3+ Tregs, and CD8+/FOXP3+ ratios in residual tumors in > 100 TNBC patients following chemotherapy, finding that elevated CD8+/FOXP3+ ratios post-chemotherapy were associated with improved clinical efficacy (104).

Treg cells have been shown to correlate positively with PD-L1 expression in TNBC (108, 110). One study found that a high number of Tregs was significantly associated with elevated PD-L1 levels (111). These findings suggest that PD-L1 and Tregs may work synergistically by participating in the same molecular pathway, and that the upregulated expression of PD-L1 and Tregs appears to promote tumor immune evasion (112).

### 3.2 Exhausted T lymphocytes

T-cell exhaustion and functional impairment of these cells in the TME is a defining feature of many cancers (113). Although tumor-infiltrating cytotoxic CD8+ T cells may effectively suppress tumor growth, CD8+ T cells can become ‘exhausted’ or ‘dysfunctional’ within the tumor. One of the hallmarks of dysfunctional T cells is an increase in certain inhibitory receptors, including PD-1, Tim-3, CTLA-4, and LAG-3 (114). One study found that CD39 ectoenzyme expression is a reliable marker of exhausted T cells in cancer (115). Exhausted T cells gradually lose their capacity to proliferate, to produce cytokines, and to lyse following chronic antigen exposure (114).

Guo et al. found that T-cell exhaustion in primary TNBC tumors correlates with longer survival and may be a prognostic factor in TNBC (116). Bottai et al. reported similar results, finding that concurrent infiltration of LAG-3+/PD-1+ TILs was associated with improved survival (117). In that study, compared to normal breast tissues, tumor tissues exhibited a marked decrease in CD8+ T cells and a marked increase in the proportion of CD4+ and CD4+ and CD8+ T cells expressing PD-1 and CD39. DFS was inversely associated with the presence of PD-1+CD8+ and CD3+CD8+ T cells in the cancer tissue. Positive associations were detected between peripheral PD-1+CD4+ T cells and PD-1+CD4+, PD-1+Cd8+, and CD39+CD8+ T cells in cancer tissue, and between CD39+CD4+ and CD39+D8+ T cells in peripheral and cancer tissue. These findings suggest that TNBC tumor cells may synergistically utilize the CD39 and PD-1 inhibitory pathways to escape the host immune response, thus leading to poor survival (118).

Bossio and colleagues examined tumor-infiltrating CD39+CD4+ T cells obtained from patients with BC, finding that these cells exhibit features of exhaustion while retaining the capacity to produce effector cytokines (119). In that study, those cells were detected in tumors, metastatic lymph nodes, and—in lower amounts—in non-cancerous breast tissue located adjacent to the tumor. Crucially, these cells were not present in non-metastatic lymph nodes or blood. These findings indicate that CD39 is a biomarker of conventional CD4+ T cells, with both depletion and cytotoxic potential.

## 4 The importance of the stroma: cancer-associated fibroblasts

The TME includes different populations of stromal cells, the most common being CAFs, which are characterized by the CD34+/PDGFRβ^+^/PDGFRα^+^/CD146^−^ phenotype. Several different CAF subtypes have been identified (including CAF-S1 to CAF-S4), based on six activation markers, as follows: fibroblast activation protein (FAP); PDGF receptor β (PDGFRβ); fibroblast-specific protein-1 (FSP-1); CD29; caveolin-1 (CAV-1) and *α*-smooth muscle actin (ASMA). In this latter subtype (ASMA), myofibroblastic CAFs (myCAF), CAF-S1, and CAF-S2, are the most common subtypes seen in TNBC (120). Several studies have demonstrated a direct association between PDGFRβ^+^ fibroblasts and worse survival outcomes (OS and PFS) in solid tumors (including non-small cell lung cancer, pancreatic cancer, and breast cancer) due to their pro-tumor and immunosuppressive properties (**Table 1**). Proteins secreted by CAFs, such as matricellular SPARC (expressed in 88% of CAFs) and matrix metalloproteinases (MMP), enable the mobility and invasiveness of TNBC cells by inhibiting cell adhesion (37). Another protein produced by CAFs (biglycan) has been associated with lower amounts of T CD8+ cells and worse prognosis in TNBC (39). High expression of CAF genes is also associated with cytotoxic T lymphocyte (CTL) dysfunction in TNBC, and generally considered an exclusion criterion in studies involving TNBC patients. CAF-S1 can recruit CD4+ CD25+ T lymphocytes by secreting CXCL12, which promotes their maturation into FOXP3^high^ (regulatory T cells) while they are retained in the TME by PD-L2, JAM2, and OX40L (40). Treg infiltration is also stimulated by CD73, a protein that is highly accumulated in CAF-S1 (42). In addition to Tregs, CAFs also recruit other immunosuppressor cells, including pulmonary fibroblasts, which activate complement signaling in an inflamed environment following chemotherapy (doxorubicin). In turn, this activation attracts MDSCs to the metastatic niche, thereby contributing to T cell dysfunction (121). MDSCs, in turn, activate CAFs that express CXCL16, a monocyte chemoattractant, which creates a positive feedback loop (43). Cytokines such as IL33, which are upregulated in CAFs, reprogram the immune system toward type 2 immunity, which also promotes lung metastasis in BC (44). Murine TNBC models have shown that CAF activates the CXCL12-CXCR4 axis to recruit monocytes and promote differentiation into lipid-associated macrophages (LAM) STAB1+ TREM2^high^, which are capable of inhibiting T cell activity and proliferation (45).

The events described above also lead to immunotherapy resistance in BC. Breast cancers that express high levels of Endo180 receptor (Mrc2) mRNA, which is expressed in myCAFs, are less sensitive to anti-PD-L1 and anti-CTLA4 ICIs in murine models and in human BC tissues. This decreased sensitivity is due to the “cold tumor” effect that CAFs confer, caused by the recruitment of Tregs via TGFβ, MDSCs via complement signaling, and CXCL16 and CTL exclusion (46).

The key role played by CAFs in TNBC opens the door to new therapeutic strategies that target CAFs, such as fibroblast grow factor receptor (FGFR) blockade with erdafitinib. This treatment can increase T cell infiltration by inhibiting the proliferation and migration of CAFs through negative regulation of the MAPK/ERK pathway, thereby decreasing the secretion of vascular cell adhesion molecule-1 (VCAM-1) (122).

## 5 Immunosuppresive signaling pathways in TNBC

As we have seen, the cells in the TME play an important role in the development of TNBC. However, the disruption of constitutive signaling pathways related to physiological processes also plays an important part (123). To date, the pathways that have received the most attention are those with a high mutational load, such as MAPK, ERK, and Akt/mTOR (124). Other pathways include alterations in the expression of molecules such as MUC-1, and alterations in the Notch pathway receptors and ligands and in the IFNγ-JAK-STAT axis. These pathways affect cell growth, the cell cycle, and the vascular or extracellular matrix remodeling factors involved in proliferation, apoptosis inhibition, dissemination, and angiogenesis. These molecules also modulate the immune system by promoting an immunosuppressive environment (**Table 2**) (146, 147). Consequently, these molecules are considered potential targets for new therapies in TNBC (148).

**Table 2:** Molecular targets and possible therapeutic strategies by approaching the signaling pathways involved in immunosuppression.

Therapeutic strategy	Target	Signaling pathway	Result	Ref(s)
γ-secretase inhibitor (GSI)	γ-secretase	Notch	Reduction in colony formation through EGFR downregulation	(125)
γ-secretase inhibitor (GSI) DAPT	γ-secretase	Notch	Reduction in prometastatic cytokines IL-8 and CCL5 production by CAFs	(126)
shUSP9X and siUSP9X	USP9X	Notch	Inhibit Notch activation through blocking multiprotein complex with TRB3	(127)
ADAM siRNA	ADAM10	Notch	Cell cycle arrest, less proliferation, and an increased sensitivity to chemotherapeutic agents	(128)
CTX014	Jag1	Notch	Reduction of tumor growth and MDSCs recruitment through Arg and iNOS inhibition	(129)
miR-598	Jag1	Notch	Apoptosis of TNBC cells, less invasiveness and lymph node metastasis	(130)
siRNA 12634	Syndecan-1	Notch/IL-6-STAT3	Decrease of immunosuppressive cytokines and growth factors	(125)
αJag1	Jag1	Notch	Inhibition of CSC proliferatiton and brain metastasis formation	(131)
anti-JAG1 single-chain variable fragments	Jag1	Notch	Activation of cytotoxic T cells	(132)
hsBCL9CT-24	BCL9/β-catenin	WNT	Cytotoxic T cell and DC infiltration and reduction of Tregs	(133)
WAV939	Tankyrase-1(*TNKS)*	WNT	Reduction of EMT factors through β-catenin degradation	(134)
miR-381	CTNNB1, RhoA, ROCK1, and c-MYC genes	WNT	Decrease in migration and invasiveness of TNBC cells	(135)
miR200c	TDO	Kynurenine	Reduction in EMT and immunosuppressive markers	(136)
1-MT and EPA	IDO	Kynurenine	Apoptosis and cell cycle arrest of TNBC cells and reversion of T cell exhaustion	(137, 138)
Indiximod	IDO	Kynurenine	Decrease in TNBC cell viability	(139)
CPT-SS-NLG919 (CN)	IDO	Kynurenine	Promotion of DC maturation, decrease in immunosuppressive cytokines, Tregs, and tryptophan consumption	(140)
LY3381916	IDO	Kynurenine	Increase in CD8+ T cells	(141)
GO-203	Muc-1	IFNγ	Overcome DNA damage resistance in TNBCs treated with carboplatin	(142, 143)
α-IFNGR1	IFNGR1	IFNγ	Reduction in tumor growth and metastasis	(144)
Bazedoxifene	GP130/IL-6 receptor	IL6-STAT3	TNBC cell proliferation and migration blockade	(145)

TRB3, tribbles homolog 3; GSI, γ-secretase inhibitor; EGFR, epidermal growth factor receptor; CAF, cancer associated fibroblast; Jag1, Jagged 1 protein; MDSCs, Myeloid Derived Suppressor Cells; Arg, Arginase; iNOS, inducible Nitric Oxide Synthase; CSCs, Cancer Stem Cells; DCs, Dendritic Cells; Tregs, Regulatory T lymphocytes; EMT, Epithelial to Mesenchymal Transition; TDO, Tryptophan 2-3 dioxygenase; IDO, Indoleamine dioxygenase; IFNγ, Interferon γ; 1-MT, 1-Methyl-L-tryptophan; EPA, Epacadostat; IFNGR1, Interferon gamma receptor 1.

### 5.1 Notch

Notch signaling is a conserved juxtracrine signaling pathway involved in some biological processes, including cell development, cell-fate acquisition, and cell proliferation, maintenance, differentiation, and death (149).

The notch pathway is closely linked to breast development and the onset of BC due to its involvement in cell proliferation processes, metastasis, and treatment resistance (150). Yang et al. classified patients with TNBC into high and low risk groups based on survival outcomes, immune response, and tumor mutational burden (TMB). They then compared these groups, finding that Notch signaling was enriched in the high-risk group (151). The different association between the Notch receptors and the pathophysiology of BC depends on the specific receptor. For example, the Notch4 receptor is more closely associated with TNBC than other types of BC. Wang et al. found that Notch4 was overexpressed in > 50% of TNBC cases, and this overexpression was also associated with larger tumor size, lymph node involvement, and recurrence (152). Notch1 has been associated with lymph node metastasis and Notch2 with other metastatic locations. Studies have shown that an increase in Notch1 and Notch3 is associated with lower DFS in triple-negative and HER-2+ tumors while blockade of Notch 2 and 3 can reduce tumor growth and the frequency of tumor cell initiation (153–155).

The molecular mechanisms involved in the pathogenesis of BC in relation to Notch signaling are not entirely clear. However, Notch1 has been shown to produce cyclin B1, Bcl2, and Bcl-XL through NFκB signaling, thus promoting cell cycle progression and cell proliferation while also inhibiting apoptosis. Notch blockade with γ-secretase inhibitors (GSI) can reduce colony formation through the downregulation of epidermal growth factor receptor (EGFR) signaling (125, 156). Cheng et al. found that TNBC tumors that overexpress ADAM10—a key enzyme in Notch activation—have a worse response to NAC, with poorer OS outcomes. In that study, by contrast, *in vitro* downregulation of Notch (through ADAM10 knockout cells) increased the 50% inhibitory concentration of paclitaxel and adriamycin, leading to cell cycle arrest and interruption of proliferation (128).

The role of Notch signaling in tumor immunity has been widely studied. The aberrant activation of Notch1 and 4 is inversely correlated with HLA gene expression, which drives T cell exclusion, and associated with high recurrence rates in estrogen receptor negative BC (157). Notch1 promotes the production of proinflammatory cytokines such as CCL2 and IL-1β, which improves the recruitment of tumor-promoting TAMs to the TME. USP9X is a deubiquitinase that forms a multiprotein structure with the pseudokinase tribbles homolog 3 to activate Notch signaling. Jaiswal et al. showed that blocking USP9X decreased tumor inflammation and growth and restored the antitumor immune response (127). Other studies have shown that GSI decreases IL-6 and IL-8 (both associated with poor outcomes in TNBC) and reduces cell migration and invasiveness in TNBC cell cultures (125, 126). Liubomirski et al. showed that co-culture models (TNBC cells with CAFs) stimulated with TNFα and IL-1β activate the p65 subunit of the NFκB pathway, leading to Notch1 signaling. As a result, CXCL8 and—to a lesser extent CCL5—are released by the CAFs. Both proinflammatory cytokines showed the capacity to increase the metastatic capacity of TNBC cells, both *in vitro* and *in vivo* (126).

The cytokines IL-1β and CCL2 have been associated with Notch signaling, but they also appear to attract MDSCs to promote immune evasion and resistance to ICIs (158). Studies have shown that Notch blockade with GSIs reduces the percentage of M-MDSC, leading to the generation of G-MDSCs with lower immunosuppressive capacity, which in turn increases antigen presentation and decreases blockage of dendritic cell signaling pathways and T lymphocyte activation (159).

#### 5.1.1 Jagged 1

Each ligand or ligand-receptor combination in Notch signaling has different effects on the oncologic process. Some Delta-like ligands (DLL) are involved in antitumor immunity. For example, DLL1 promotes differentiation of M1-TAMs while DLL1-Notch 4 facilitates T naïve polarization into Th1 and Th2 (160, 161). DLL3 and DLL4 are associated with tumor-promoting phenomena and poor prognosis (162, 163). Of all the Notch pathway ligands, Jagged-1 (Jag-1) stands out for its involvement in the pathogenesis of BC, especially TNBC. Strati et al. found that the JAG1 cytoplasmatic protein was expressed in 43% of tumors in a large sample (n=333) of patients but nuclear expression of this protein was detected in only 17% (164). Speiser et al. found that JAG1 expression was associated in TNBC patients with nodal involvement and larger tumor size, correlating with worse prognosis and survival (165).

Jag1 participates in different mechanisms involved in tumorigenesis, including tumor growth, maintenance of CSCs, angiogenesis, invasion, metastasis, and immune evasion. In TNBC cell models, Notch signaling through the Jag-1 ligand promotes AKT phosphorylation by a mechanism dependent on the NFκB pathway, through IKKα, the kinase that phosphorylates and inactivates IKBα (the inhibitory subunit of NFκB), thus leading to an increase in oxidative metabolism. The positive regulation of NFκB increases expression of the antiapoptotic gene cIAP-2, which improves survival of CSCs (166).

The Jag1-Notch3 pathway promotes VEGF secretion, which in turn promotes angiogenesis (167). Liu et al. found that JAG1 expression was higher in MDA-MB-231 bone cells (MDA-MB-231B) than in MDA-MB-231 cells, with the former being the more aggressive subtype. This high expression was accompanied by exosomes with high levels of long, noncoding RNA (lncRNA) MALAT1, thus promoting increased angiogenesis (VEGF and CD31 expression), migration and invasion in MDA-MB-231B cells, and a decrease in apoptosis (168, 169). MALAT1 also affected the monocyte-macrophage activity in the premetastatic niche via downregulation of miR-26a-5p, which, in turn, increased expression of Jag-1 in monocyte-macrophages, altering their adhesion, migration, and differentiation (170).

The factors that cause aberrant activation of the Notch pathway are also observed in patients with TNBC with a poor prognosis. For example, suberoylanilide hydroxamic acid, a histone-deacetylase blocking agent, augments Jag-1, 2, Hes1, and c-Myc and improves EMT (171). The bromodomain and extra terminal (BET) protein BRD4 is an upstream regulator of JAG1 that improves mRNA and protein levels, leading to the migration and invasion of TNBC cells and MAT inflammation via the release of IL-6 and T-bet+ TIL infiltration, eventually resulting in distant metastasis. BDR4 is also associated with an increase in the ki-67 index; therefore, it plays a key role in the proliferative process and in tumor development (172).

In the context of tumor immunity, TNBC cells secrete factors that induce Jag-1 expression in MDSCs through the NFκB-p65 pathway. CTX014, an inhibitor of Jag-1, can limit tumor growth and recruitment of MDSCs by blocking Arginase1 and iNOS. This effect also facilitates the infiltration of reactive-CD8+ T cells and improves the outcomes of T cell therapy (129). In cases with minimal residual disease, Jag1 allows for the maintenance of CSCs and immune cell homeostasis in MAT and EMT, all of which increase the risk of recurrence (173).

In light of the information described above, it is clear that Jag-1 is an important potential therapeutic target. In this regard, Han et al. found that levels of miR-598—the main target of which is Jag1—are low in TNBC tissues. The presence of low levels of this miRNA implies a greater propensity for invasiveness and nodal metastasis. Those authors also found that TNBC cell viability could be reduced through apoptosis by inducing ectopic expression of miR-598 (130). Another study also observed the same effect through inhibition of Jag1 with siRNA in MDA-MB-231B cell cultures (174). In rat models, administration of Jag1 antibodies can inhibit proliferation of CSCs, reduce the growth of BC spheroids, and prevent brain metastasis without any treatment-related toxicity (131). Silva et al. generated a chimeric antigen receptor T cell (CAR-T) anti-Jag1 therapy capable of activating cytotoxic T cells in Jag1 expressing cell models (132).

### 5.2 Wnt pathway

The Wnt signaling pathway regulates many cellular developmental processes such as embryogenesis, cell fate, stem cell pluripotency, tissue homeostasis, regeneration, and differentiation. In this signaling pathway, there are several different models including Wnt-Ca^2+^ and the Wnt planar cell polarity pathways. However, the most studied pathway is classical pathway (Wnt-βcatenin) (175, 176).

The WNT pathway is involved in various biological processes and has also been linked to several types of cancer, including TBNC. A meta-analysis that included a total of 1,878 cases of BC found that aberrant activation of Wnt-βcatenin was especially common in TNBC compared to other types of breast cancer (177). Activation of Wnt-βcatenin has been shown in phenotypes associated with lung and brain metastases due to fibronectin-directed migration, F-actin organization, and invasion phenomenon. Apart from β-catenin, other proteins of the Wnt pathway such as cyclin kinase 14 (CDK14) are also elevated in TNBC (177, 178). Gene expression profiles of TNBC reveal high levels of Wnt genes such as Wnt10A (179).

Although the effects of Wnt/β-catenin on the recruitment of TILs remain controversial, Ma et al. found that overexpression of β-catenin was associated with high levels of TILs (180). This pathway appears to influence immunosuppression in several different ways. WISP1, a tumor-derived Wnt-inducible protein, promotes type 2 immunity polarization through inhibition of IL-12 (181). Wnt5a activation is associated with secretion of cytokines such as PDGF-AA and CCL2, which leads to M2 polarization (182). The proteins, BCL9 and BCL9L—βcatenin coactivators—promote tumor cell growth, migration, and metastasis, and have been inversely correlated with CD8+ T cell infiltration. Wang et al. (133) found that inhibition of these proteins inhibited tumor growth and also increased the number of DCs and the effectiveness of ICIs while also decreasing Tregs (133). Gong et al. showed that stimulation of APC (a Wnt inhibitor protein) through m6A methylation transferases (METTL14 and ZC3H13) increased the infiltrating levels of several cell types, including DCs, CD8+ and CD4+ T cells, M1-TAMs, while also decreasing levels of Tregs (183). Although Wnt-mediated immune evasion has been associated with distinct immune checkpoint and markers of exhaustion expression (including PD-L1, CTLA-4, LAG3, CD86, ICOS, ICOSLG and TNFSF9) (184, 185), a high density of CD163+ myeloid cells and anti-PD1 treatment resistance has observed in overactivated Wnt-PARPγ signaling (186). Thacker et al. discovered a protumorigenic NK cell subpopulation, exclusively observed in TNBC, that was capable of activating CSCs and reducing the effectiveness of ICIs through the Wnt pathway (187).

Wnt also plays a role in treatment resistance in TNBC. β-catenin is associated with a worse response to chemotherapy; however, genotoxic treatments, such as radiotherapy and chemotherapy, can activate the Wnt-βcatenin pathway independent of the LRP (LDL receptor–related protein) and FZL (Frizzled protein) ligands. The DNA damage caused by these treatments activates OTULIN, a deubiquitinase that blocks β-catenin degradation (188, 189). However, Shetti et al. showed that the combination of the Wnt inhibitor XAV939 and paclitaxel can decrease β-catenin expression in three TNBC cell lines, thus inhibiting EMT and angiogenic markers and increasing E-cadherin mRNA and protein levels, which antagonize EMT (134). MicroRNAs can also be used as a therapeutic strategy in TNBC. For example, one study found that miR-27a-3p was highly expressed in TNBC and attenuates GSK3β and inactivates Wnt-βcatenin, resulting in a decrease of cell proliferation and migration (190). In murine models, it has been shown that MiR-381 can decrease the migratory and invasive potential of MDA-MB-231 cells, thus reducing lung metastasis and improving OS (135).

Given this background, it is clear that more studies are needed to better elucidate the mechanisms that implicate Wnt signaling in TNBC in order to determine how to best take advantage of its therapeutic potential.

### 5.3 IFN-γ signaling pathway

Numerous innate and adaptive immunity cells generate IFN-γ in the TME. The production of IFN-γ is generally mediated by a range of different cell types, including T, NK, iNKT, T reg, γδ T, and B cells (191). IFN-γ has two opposing effects: antitumor and protumor (192). IFN-γ signaling has diverse biological functions, mainly associated with host defense and immune regulation, including antibacterial and antiviral defense, apoptosis, inflammation, the cell cycle, and innate and acquired immunity. The main role of IFN-γ is to regulate MHC class I molecules to assist in antigen priming and presentation on antigen-presenting cells (193). IFN-γ also has protumor functions in several types of cancer, including BC (194).

The pleiotropic effects of IFN in the TME are complex. The overall impact on tumor growth depends on the balance between antitumor IFN-γ signaling (tumor cell destruction, effector function, cell migration, immune cell proliferation, and antigen presentation) and protumor IFN-γ signaling (immunosuppression, angiogenesis, and tumor cell proliferation) (191). Due to its effective antitumor activity, IFN-γ is considered a potential immunotherapeutic agent against cancer (195).

The main IFN-mediated signaling pathway is the JAK-STAT pathway. Canonical IFNG signaling is initiated by the binding of the biologically-active form of IFNG to its two subunit receptors, IFNGR1 and IFNGR2. These subunits interact intracellularly with JAK family kinases (JAK1 and JAK2, respectively), leading to the phosphorylation, activation, and dimerization of STAT1 transcription factors. In turn, phosphorylation of STAT1 leads to its translocation to the nucleus where it binds to the consensus gamma-activated sequence (GAS) sites in the promoters of several target genes and activates their expression (196).

Tumor cells share a common mechanism to evade the immune response, including amplification of inhibitory molecules of the IFN-γ pathway and downregulation and loss of IFN-γ receptor and downstream signaling intermediates. Interruption of IFN-γ signaling in tumor cells may enhance tumor growth and affect the efficacy of ICIs (197). Prolonged treatment with IFN-γ has been reported to suppress gene expression in BC cells (198).

Singh et al. found that constitutive IFN-γ signaling induced by depletion of the tumor suppressor transcription factor Elf5, together with its ubiquitin ligase FBXW7, increases the number of highly immunosuppressive neutrophils in the TME, which in turn promotes tumor growth and metastasis, leading to a worse prognosis of TNBC (144). Another study showed that aging can systematically reduce IFN-γ signaling in patients with TNBC and thus limit the efficacy of ICB therapy (199).

#### 5.3.1 Interleukin-6 and STAT3

IL-6 is a soluble mediator with a pleiotropic effect on immune response, inflammation, and hematopoiesis (200). IL-6 is released by a wide range of immune and non-immune cells, such as T and B lymphocytes, endothelial cells, monocytes, keratinocytes, fibroblasts, and adipocytes (201). Aberrant expression of IL-6 is present in many types of cancer and associated with poor clinical outcomes and metastasis (202).

The two molecules implicated in the biological activity of IL-6 are IL-6R (also known as IL-6Rα, gp80, or CD126) and gp130 (also known as IL-6Rβ or CD130). Binding of IL-6 to mIL-6R (membrane-bound IL-6R), generates homodimerization of gp130 and subsequent formation of a functional high-affinity receptor complex of IL-6, IL-6R and gp130 (203). Its principal signaling mechanism involves activation of the transcription factor STAT3. The IL-6/IL-6R/gp130 complex activates JAK phosphorylation, followed by STAT3 phosphorylation, which forms a homodimer and translocates to the nucleus to activate transcription of the target gene (204).

The IL-6 signaling pathway is frequently activated in breast cancer and can promote tumor cell growth while also suppressing the antitumor immune response (205). The presence of elevated serum levels of IL-6 has been associated with poor prognosis in BC patients (206). Labovsky et al. found that both IL-6 and its receptors were overexpressed in early breast cancer when compared to expression levels in normal breast tissues (207). IL-6 is upregulated in the serum of patients with advanced and/or metastatic BC (208). Increased IL-6 secretion and STAT3 phosphorylation promote the development and progression of BC, leading to worse survival outcomes (209).

STAT3 is overexpressed and constitutively stimulated in TNBC and strongly associated with disease onset, progression, metastasis, resistance to chemotherapy, and poor survival outcomes (210). Tzeng et al. suggested that the Src/STAT3 signaling pathway is implicated in multidrug resistance in TBNC cells (211). STAT3 interacts with other oncogenic transcription factors (e.g., GLI1) to promote tumor aggressiveness in TNBC (212). STAT3 is also involved in hypoxia-induced chemoresistance in TNBC. Hypoxia can regulate STAT3 activation to promote progression and metastasis (213). Ma et al. showed that estrogen-related receptor α (ERR-α), a key target gene of STAT3, could induce metastasis in TNBC (214).

A recent study by Morrow et al. found that high STAT3 expression in tumor-associated stroma was significantly associated with lower survival in TNBC patients. In that study, the elevated stromal STAT3 phenotype was characterized by a dense, immunologically cold stromal EMT and a differential gene expression profile (215). Data from multiple studies suggest that IL-6 and STAT3 may be potential molecular targets for the treatment of TNBC (125, 145, 205, 210, 216).

### 5.4 Mucin 1

Mucin 1 (MUC1) is a glycoprotein involved in the metastasis and invasion of several different tumor types. MUC1 is located at the apical edges of normal epithelial cells, such as those in the breast, lung, pancreas, and gastrointestinal tract, where it responds to stress signals, such as inflammation and cell damage (217). Due to the multifaceted role of MUC1, MUC1-C can be considered a potential therapeutic target for TNBC (218–220).

MUC1 has two subunits that form a heterodimer at the apical cell membrane. The N-terminal subunit of MUC1 (MUC1-N) is located beyond the glycocalyx in a mucosal gel that acts as a physical barrier. The C-terminal transmembrane subunit of MUC1 (MUC1-C) is activated by loss of homeostasis, which leads to remodeling, inflammatory remodeling, and repair responses associated with wound healing (217). MUC1-C acts as an oncoprotein by interacting with several effectors associated with distinct tumor cell characteristics (221).

Chronic activation of MUC1-C due to prolonged inflammatory cycles of epithelial cell damage and repair leads to cancer progression (222). MUC1-C has an intrinsically disordered cytoplasmic domain, acting as a node to integrate different signaling pathways (223). MUC1-C activates the inflammatory transcription factors STAT3 and NF-κB in self-inducible loops, which in turn potentiate MUC1-C expansion (222). MUC1-C also promotes cell survival in TNBC by regulating the anti-apoptotic gene BCL2A1 through NF-κB p65 (224).

MUC1 can stimulate activation of the PI3K/AKT signaling pathway, ERK, and receptor tyrosine kinases to promote cancer cell growth (225). Hiraki et al. demonstrated that MUC1-C activates the MEK/ERK and PI3K/AKT pathways and is involved in autophagy. Given these findings, MUC1-C represent a potential target to reverse resistance in TNBC (226).

MUC1-C chronically activates IFN-γ through the JAK1/STAT1/IRF1 pathway and induces IDO1 and cyclooxygenase2 (COX2)/PTGS2 effectors, which play an important role in immunosuppression. Yamashita et al. found that MUC1 was associated with CD8+ T-cell exhaustion and dysfunction in TNBC (142). In another study, the same group showed that MUC1-C activates the type I IFN pathway and induces resistance to DNA damage in TNBC cells. In response to carboplatin, targeting MUC1-C helped to overcome DNA damage resistance in the treatment of TNBC (227).

MUC1-C chronically activates IFN-γ through the JAK1/STAT1/IRF1 pathway and interacts with IDO1 and COX2/PTGS2 effectors, which are involved in immunosuppression. Kufi et al. found that MUC1 was associated with CD8+ T-cell exhaustion and dysfunction in TNBC (221). MUC1 activates PD-L1 transcription through recruitment of MYC and NF-κB p65 to the PD-L1 promoter, thus enhancing immune evasion (223). MUC1 also interacts directly with MYC to activate the NuRD complex and mediate the regulation of estrogen receptors in TNBC cells (228). MUC1-C drives lineage plasticity in TNBC cell progression by inducing EMT to promote the expression of the transcription factors ZEB1, TWIST1, and SNAIL (229).

MUC1-C activation and stem cell status have been implicated in resistance to DNA damage and immune evasion in TNBC (143). A recent study shows that MUC1-C induces PBRM1, and, in turn, the MUC1-C/PBRM1 complex enhances chromatin accessibility and activation of IFN-stimulated genes (ISG), which are involved in DNA damage resistance, chronic inflammation, and immune evasion (230).

Disease progression in TNBC is influenced by metabolic reprogramming. MUC1 expression can trigger metabolic reprogramming of glutamine utilization. Goode et al. found that aminotransferase could be a potential therapeutic target in TNBC, especially in cases with MUC1 overexpression (231). MUC1-C upregulates genes that encode HK2, GLUT1, and PGAM1 (230).

## 6 Metabolomics in TNBC

Metabolic reprogramming is one of the processes that determines the pathogenesis of TNBC. In recent years, the application of metabolomics to characterize TNBC has revealed alterations in the pathways and metabolites of tumor cells involved in numerous tumor-promoting processes: hypoxia, proliferation, survival, immune evasion, and treatment response, among others.

TNBC can be classified according to is metabolomic profile. Xiao et al. (232) described three different subtypes, as follows: C1, characterized by a high ceramide and fatty acid content; C2, which presents an increase in oxidation and glycosyl transfer metabolites; and C3, characterized by relatively low levels of metabolic dysregulation. Those authors also described groups C2 and C3 as having a basal-like immunosuppressed transcriptomic subtype (232). Gong et al. proposed three other metabolic pathway-based subtypes (MPS), as follows: MPS1 (or lipogenic), due to hyperactivation of lipid metabolism; MPS2 (or glycolytic), in which both carbohydrate and nucleotide metabolism are upregulated; and MPS3, a mixed subtype with partial metabolic pathway alterations (233). The metabolomic characterization of TNBC could also be used for diagnostic purposes. Li et al. (234) found 77 metabolites from plasma samples whose levels differed significantly between TNBC patients and healthy donors. Those authors also established a panel of metabolic signatures that correlated closely with 5-year survival outcomes. Jin et al. used metabolites (D-dimer, CEA, L5CH, CA15.3, glutamine, and ornithine) to distinguish TNBC from other types of BC (235, 236). Eghlimi and colleagues designed a biomarker panel based on choline and glycerophospholipid metabolism and sphingolipid signaling to identify early TNBC in plasma samples (235, 236).

Metabolomics is also involved in assessing treatment response. For example, it can be used to detect alterations in the metabolism of certain amino acids (e.g., arginine, proline, glutathione, and β-alanine) associated with doxorubicin resistance (237). Carneiro et al. showed that an increase in phosphocreatine, taurine, and NF-κB pathway signaling, together with a decrease in ERK pathway signaling, glycolytic and glutaminolytic activity and nucleotide depletion, improved cisplatin resistance in MDA-MB-231 cells (238). Response to NAC is also associated with the metabolic profile. One study found that overexpression of oxidative phosphorylation was associated with worse response and inhibition of this phosphorylation and CDK4 improved response rates in TNBC (239). He et al. proposed a model to predict sensitivity to NAC based on three proteomic pathways (Gly, Ser, Thr/Val, Leu, Ile/Ala, Asp, Glu) that differ among partial responders, complete responders, and stable disease groups (240). Zapater-Moros et al. found differences in the long-chain fatty acid pathways in plasma obtained from responders vs. non-responders (241). Tryptophan catabolism is also a biomarker of response to NAC; Salvador-Coloma et al. found that an increase in chlorokynurenine with a decrease in indoleacetic acid is associated with better response (78). In turn, treatment response is associated with IDO expression due to the relationship between this enzyme and immunosuppressive cells such as MDSCs and metabolites such as anthranilic acid (AA) and 3-hydroxylanthranilic acid (3HAA) (78, 242).

Metabolism can polarize macrophages through the M1 or M2 phenotype (243). Tumor-educated macrophages (TEM) present alterations in polyamines, lipid metabolism, and adenosine accumulation. These alterations have been observed by culturing THP-1 derived macrophages with conditioned media from the MDA-MD-231 cell line, which contains immunoregulatory metabolites (244). This finding suggests that immunoregulatory metabolites could be a potential biomarker of response to immunotherapy in TNBC. However, more metabolomic studies are needed to better determine the association between metabolism and response to ICIs in TNBC.

### 6.1 Tryptophan metabolism

Tryptophan metabolism occurs through the serotonin pathway in which tryptophan hydroxylase (TPH1) synthesizes 5-hydroxytryptamine (5-HT; also called serotonin) from tryptophan. Metabolism can also occur through the kynurenine pathway involving two enzymes, tryptophan 2-3 dioxygenase (TDO) and IDO. These two enzymes catabolize L-tryptophan, giving rise to metabolites such as kynurenine. This has a direct effect on the immune system since this amino acid is essential for the correct functioning and proliferation of cytotoxic T lymphocytes. The metabolites resulting from the degradation of tryptophan lead to apoptosis of CD8+ T lymphocytes. This phenomenon plays a key role in the TME since it promotes immunosuppression and the escape phase (245).

In breast cancer, the metabolic pathway that has received the most attention is the kynurenine pathway, although a growing number of studies have also evaluated the role of the serotonin pathway. For example, Gautam et al. found that TNBC had higher cellular levels of TPH1 mRNA compared to other types of BC (246). Those authors also found that treating MDA-MB-231 cultures with 5HT promoted invasive, angiogenic (increased VEGF levels), and proliferative properties through the 5-HT7 receptor, which activates Ras/Raf/MAPK and PI3K/Akt signaling. That study also correlated increased expression of the 5-HT7 receptor with a high proliferation index in TNBC cells, which was found to promote FOXM1, cyclin D1, and eEF2K signaling. Other studies have found that expression of the 5-HT7 receptor is a biomarker of poor prognosis in TNBC and associated with poor OS. Serotonin has also been shown to increase TPH1, thereby creating a feedback loop (246–248).

Although our main focus in this section is to describe the role of the kynurenine pathway (especially the IDO enzyme) in TNBC, it is important to discuss the role of TDO in TNBC. TDO2 (TDO gene) expression has been associated with the cancer stage, presence of immune infiltrates, disease aggressiveness, poor OS and DFS, and clinical outcomes in TNBC. TNBC cells could acquire resistance to anoikis (a programmed cell death process) mediated by TDO via NF-κB activation. Some studies have shown that inhibition of TDO not only increases sensitivity to anoikis, but also decreases the migration, invasiveness, and pulmonary dissemination of these cells. TDO2, in turn, promotes the expression of other tryptophan metabolism genes, such as IDO (249–251), kynureninase, and KMO (kynurenine 3-monooxigenase). Kynureninase and KMO degrade kynurenine giving rise to metabolites with immunosuppressive capacity, such as AA and 3HAA (242). TDO inhibition through miR-200c reduces kynurenine, which has been associated with a decrease in markers of EMT and immunosuppressive factors such as CD274, CD273, HMOX-1, and GDF15 (136).

### 6.2 Indoleamine 2-3 dioxygenase

Indoleamine 2-3 dioxygenase is an enzyme that regulates immune tolerance processes under physiological conditions. It is highly expressed in the placenta in trophoblast cells and macrophages to prevent inflammation during pregnancy. IDO is closely associated with immune evasion in cancer by depleting tryptophan and reducing the recruitment of specific T cells (252).

Studies have found that IDO is more highly expressed in patients with BC (and especially TNBC) compared to healthy people. Although IDO expression is positive for MDA-MB-231 cells, other cell lines—such as MCF-7 (positive for hormone receptors)—do not show high levels of IDO mRNA (253, 254). IDO expression is associated with low OS rates and it has been correlated to clinical stage (255): approximately 70% of stage III or higher TNBCs are IDO positive. Moreover, IDO positivity has been found in circulating microvesicles, lymph node infiltrates, and in 90% of metastatic tumor cells (256–258).

The role of IDO in immunosuppression in TNBC has been well-studied. Apart from limiting T lymphocyte activity through tryptophan deprivation, IDO also increases serum levels of the immunosuppressive acidic protein (IAP). In most cases, IDO is co-expressed with immune checkpoints, mainly PD-L1; in fact, more than 70% of PD-L1+ TNBC cells express IDO (253, 258, 259).

TNBC is a “hot” tumor (i.e., likely to trigger a strong immune response) because it is an immunoreactive tumor due to the inflamed microenvironment, which is characterized by the presence of cytokines such as IL-1, IL-2, TNFα, and IFNγ produced by CD8+ T cells, NK cells, and M1 macrophages through DC signaling and GM-CSF stimulation (260). These cytokines and COX-2 secreted by TNBC cells can promote the expression of IDO and shift the microenvironment towards type 2 immunity (261, 262). In turn, this leads to recruitment of immunosuppressive cells such as Tregs (whose presence has been correlated with IDO expression in TNBC) to the TME (263). Chen et al. evaluated tissues from 41 patients with TNBC, finding that the tumor-immune boundary was enriched in immune cells, with increased levels of IDO, PD-L1, and FoxP3 CD45+ cells (264). Immunohistochemical studies have shown that IDO+ MDSCs increase the proportion of Tregs, which is associated with tumor grade, poorer response rates, and worse prognosis (265). IDO has been shown to increase the immunosuppressive properties of MDSCs. IDO expression is stimulated, in turn, by IL-6 through a mechanism dependent on non-canonical NF-kB and STAT3 signaling (266).

For all the reasons described above, it is not surprising that many studies have been performed to evaluate the role of the IDO enzyme. In preclinical models, 1-Methyl-L-tryptophan (1-MT)—an IDO inhibitor—has been shown to restore the status of exhausted CD8+ T lymphocytes and improve their cytotoxic ability by increasing perforin production (137). The combination of 1-MT with another inhibitor (epacsdostat [EPA]) produces cell cycle arrest in the G0/G1 phase and apoptosis of TNBC cells (138). By blocking IDO with indiximod, the cell viability in TNBC is reduced through an apoptosis mechanism. Indiximod can be combined with TNFα to enhance the effectiveness of ICIs (139). CPT-SS-NLG919 (CN) and its nanoformulation (CN@PLA-HES-FA) is a novel prodrug that inhibits IDO and can promotes DC maturation, a decrease in Tregs, and tryptophan consumption, and can lower the production of immunosuppressive cytokines (e.g., IL-6, IL-13 and TGFβ) in murine models of TNBC (140). A phase I study found that the combination of an IDO inhibitor (LY3381916) with anti-PD-L1 increased recruitment of CD8+ T cells in the TME, leading to disease stabilization in cancer patients (141).

These findings demonstrate the strong involvement of tryptophan metabolism in the inhibition of the antitumor immune response, which potentially opens up new avenues to develop novel therapies for TNBC.

## 7 TNBC as a candidate for immunotherapy

TNBC is associated with mutations in multiple genes, mainly TP53 (accounting for up to 30% of cases), PIK3CA (≈ 30% of cases) and the genes BRCA1 (20%) and BRCA 2 (15%). Numerous other genes are commonly mutated in TNBC, including PIK3CA, KRAS, MET, BRAF, EGFR, and PTEN. These mutations lead to neoantigens capable of activating the immune system and increasing the immune infiltrate (123, 267–269).

Another important characteristic of TNBC is the expression of immune checkpoints such as PD-L1, which is present in 20% to 40% of cases. This overexpression has been associated with several other mutations, including PTEN and PI3K, which promote CD8+ T cell infiltration. In fact, this could explain why TNBC tumors have the highest percentage of TILs among all BC subtypes (270, 271).

TNBC is a highly immunogenic tumor. Given that tumors with CD8+ TILs and CD163+ macrophages appear to respond better to immunotherapy (272), TNBC is a good candidate for immunotherapy (ICIs or antitumor vaccines) (273–275). In recent years, multiple clinical trials have evaluated immune therapies in TNBC. In general, treatment outcomes (response and survival rates) in patients treated with immunotherapy are better than in patients who receive conventional therapies (**Table 3**).

**Table 3:** Completed clinical trials of immunotherapy in TNBC with main results.

Trial identifier	Phase	TNBC stage	Therapy	Main results	Ref
Immune checkpoint inhibitors (ICIs)					
NCT02530489	II	I-III	Atezolizumab (PD-L1) + nab-paclitaxel	pCR=46%	(276)
NCT02685059 (GeparNuevo)	II	II-III	Durvalumab (PD-L1) +/- nab-paclitaxel	ORR=53.4% Durvalumab vs 44.2% placebo pCR=61% Durvalumab vs. 41.4% placebo	(277)
NCT04213898	II	II-III	Camrelizumab (PD-1)+ nab-paclitaxel+ epirubicin	pCR=64.1% ORR=89.7%	(278)
NCT03036488 (KEYNOTE-522)	III	II-III	Paclitaxel + carboplatin +/- pembrolizumab (PD-1)	pCR=64.8 pembrolizumab vs. 51.2% placebo	(279)
NCT0142379 (I-SPY2)	II	II-III	Taxane + anthracycline +/- pembrolizumab (PD-1)	pCR=60% pembrolizumab vs. 22% placebo	(280)
NCT03616886 (SYNERGY)	II	IV	Paclitaxel+ carboplatin+ Durvalumab (PD-1) +/- Oleclumab (CD73)	Median PFS=5.9m oleclumab vs 7m without oleclumab	(281)
NCT02447003 (KEYNOTE-086)	II	IV	Pembrolizumab (PD-1)	ORR= 5.3% PFS=2 m OS=9 m	(282)
NCT02730130	II	IV	RT+ pembrolizumab (PD-1)	ORR=17.6% (3 CR, 1 SD, 13 PD)	(283)
NCT02768701	II	IV	Cyclophosphamide (T reg depletion) prior to pembrolizumab (PD-1)	ORR=21% PFS=1.8 m	(284)
NCT03121352	II	IV	Carboplatin+Paclitaxel+ Pembrolizumab (PD-1)	ORR=48% (2 CR, 11 PR, 8 SD) DOR=6.4 m PFS=5.8 m, OS= 13.4 m	(285)
NCT02513472 (ENHANCE 1)	Ib (1^st^ line)/II (2^nd^ – 3^rd^ line)	IV	Eribulin+ Pembrolizumab (PD-1)	ORR=25.8% Ib vs. 21.8% II	(286)
NCT02819518 (KEYNOTE-355)	III	III-IV	ChT +/- pembrolizumab (PD-1)	Median OS=23 m pembrolizumab vs. 16.1 m placebo (CPS>10)	(287)
NCT03945604	Ib	IV	Camrelizumab (PD-1)+ Apatinib (VEGF2 tyrosine kinase) + Fuzuloparib (PARP1)	ORR=6.9% PFS= 5.2 m 1 Year OS= 64.2%	(288)
NCT04303741	II	III-IV	Eribulin+ Camrelizumab (PD-1) + apatinib (VEGF2 tyrosine kinase)	ORR= 37% PFS= 8.1 m	(289)
NCT0412996 (FUTURE-C- Plus)	II	III-IV	Famitinib (angiogenesis inhibitor) + nab-paclitaxel + Camrelizumab (PD-1)	ORR= 81.3% PFS= 13.6 m	(290)
WJOG9917B (NEWBEAT)	II	IV	Nivolumab (PD-1) + bevacizumab (VEGF)+ paclitaxel	ORR= 59% PFS median= 7.8 m OS= 32.5 m	(291)
NCT02425891 (IMpassion 130)	II	III-IV	Nab-paclitaxel +/-atezolizumab (PD-L1)	OS= 21 m atezolizumab vs. 18.7 m placebo	(292)
NCT01633970	Ib	III-IV	Nab-paclitaxel + atezolizumab (PD-L1)	ORR= 39.4% PFS median= 5.5 m OS= 14.7 m	(293)
NCT02471846	I	III-IV solid tumors (including TNBC)	Navoximod (IDO inhibitor) + atezolizumab (PD-L1)	ORR= 9% dose-escalation phase and 11% expansion phase	(294)
Viral therapies					
NCT04185311	I	I-III	Nivolumab (PD-1) + Ipilimumab (CTLA-4) + intratumor talimogene laheparepvec (oncolytic virus)	1 pCR ((16.7%), 3 PR (50%), 1 SD (16.7%), 1 PD (16.7%) Increase of T cell infiltrate	(295)
n/a	II	IV	Stereotactic body radiotherapy (SBRT)+ herpes-simplex-virus thymidine-kinase + pembrolizumab (PD-1)	ORR=21.4% (7.1% CR, 3.6% PR, 10.7% SD) OS= 6.6 m	(296)
NCT03256344	Ib	IV (TNBC & Colorrectal)	intratumor talimogene laheparepvec (oncolytic virus) + atezolizumab (PD-L1)	ORR= 10% in TNBC patients AEs= 90% of patients	(297)
Cancer vaccines					
UMIN000014616	II	IV	Mixed 19-Peptide cancer vaccine	OS= 11.5 m (24.4 complete vaccine)	(298)
NCT01421196	II	II-III (Luminal and TNBC)	NAC EC following taxanes +/- DCV	pCR in TNBC= 50% DCV vs 30.5% non DCV). Increasing of CD8 TILS (4.48% baseline vs 6.7% after surgery)	(95)
CAR-T therapies					
NCT03060356	I	IV (TNBC & Melanoma)	RNA-electroporated c-Met directed chimeric antigen receptor (CAR) T cells	57.1% SD Increase of CD8 and CD3 T cells in TME. Decrease of p56 and Ki67	(299)
NCT04025216	I	IV (solid tumors including TNBC)	TnMUC1 targeted chimeric antigen receptor T-cells + ChT.	100% SD at day +28 T cell expansion No serious AEs	(300)

ORR, objective response rate; PFS, progression free survival; OS, Overall survival; AE, adverse events; pCR, pathologic complete response; PR, partial response; SD, stable disease; PD, progression disease; EC, Epirubicin- cyclophosphamide; DCV, Dendritic Cell Vaccine; ChT, chemotherapy; m, month.

Based on clinical studies conducted with ICIs in patients with TNBC, pembrolizumab, a monoclonal antibody targeting PD-1, is currently approved for use in both early-stage and advanced disease, demonstrating a survival benefit when combined with chemotherapy. The KEYNOTE-522 trial, conducted in patients with stage II and III TNBC, showed that adding pembrolizumab to optimal neoadjuvant chemotherapy increased the pathological complete response rate by 15%, with observed benefits in event-free survival and overall survival at 3 years (279). This benefit was seen across the entire population, regardless of PD-L1 expression status. In advanced disease, the survival benefit of adding either pembrolizumab or the anti-PD-L1 monoclonal antibody atezolizumab to first-line chemotherapy appears to be limited to patients whose tumors express PD-L1 (292, 293). In early-stage disease, ongoing studies such as the GeparDouze trial are exploring the effectiveness of neoadjuvant atezolizumab followed by adjuvant therapy (301). Other antibodies against PD-L1 (i.e., durvalumab and avelumab) have also been evaluated in different disease stages with limited benefits (277, 281). Ongoing trials in TNBC are exploring the potential synergism of ICIs with radiation therapy (abscopal effect), antibody drug conjugates (ADC) (302, 303), PARP-inhibitors (304), PI3K inhibitors and antiangiogenic drugs (290, 305). Other cell surface immunoregulatory proteins like CD276 are explored with the generation of specific or bispecific antibodies (306–309).

A high proportion of triple-negative tumors fail to respond to immunotherapy. Although PD-L1 expression and the presence of TILs are reasonable markers, we are still far from obtaining strong predictive signatures for ICIs in TNBC. It has been observed that clusters of immune cells associated with *in situ* immunity, known as tertiary lymphoid structures (TLS), appear near the tumor. These structures have been associated with the percentage of TILs, and authors such as Seow et al. have suggested that these could be used as biomarkers of good prognosis and better response to treatment (310, 311). Some studies have even proposed using TLS for treatment purposes (312). Other authors, such as Liu et al., have described a TLS subtype that expresses immunosuppressive cells and genes that are associated with worse prognosis (313). Therefore, the benefit of TLS may be related to the cellular composition of these structures. In this regard, expanding our knowledge of these structures could provide a better understanding of cancer immunology to develop new strategies to improve response rates to ICIs (314).

In addition to ICIs, several other strategies have emerged in recent years. Adoptive cell therapies, which have shown good results in hematologic cancers (315–317), are beginning to be developed for the treatment of solid tumors. However, the application of these therapies can be challenging due to an immunosuppressive TME, the absence of specific neoantigens, and associated toxicities. Beyond CAR-T cells, macrophages and NK cells have been proposed (318–320). Several clinical trials have been initiated for agnostic tumors showing high TMB that in general incorporate TNBC, but the available data are inconclusive (321–323). The use of TILs in advanced disease is also being considered (324). The phase II NeoTRACT trial is exploring the efficacy of neoadjuvant therapy with TILs in early-stage disease. Other emerging strategies include cancer vaccines, oncolytic viruses, and DCs, all of which are being evaluated in different clinical trials (NCT04105582, NCT06324240, NCT04879888) (325, 326).

One of the pathogenetic characteristics of TNBC is its ability to disseminate into sites such as the bone marrow (BM) and the central nervous system (CNS), which poses an important challenge in the treatment of advanced disease. Up to 40% of patients with metastatic TNBC will develop brain metastasis or leptomeningeal disease. Although brain disease may be treated with surgical resection or radiotherapy, the available systemic therapies consistently show limitations to penetrate the blood-brain barrier (BBB) (327). Significant activity has been observed in TNBC patients with brain disease with the specific ADCs Sacituzumab-govitecan or trastuzumab-deruxtecan. Several trials with new ADCs are exploring this specific niche (328–330).

Gene expression of disseminated tumor cells (DTC) to BM has been proposed as a possible prognostic biomarker related to the probability of metastatic relapse (331). At present, few options are available to treat BM lesions. Murine experiments have shown that agents such as cyclophosphamide are able to recruit immune cells to the bone marrow and synergize antibody therapies (332). The use of agents such as denosumab has been explored without much success (333). CDK inhibitors have been able to prolong survival and control disease progression in other BC subtypes (334, 335). However, this metastatic niche still lacks effective therapeutic options and most studies are still in the preclinical stage.

## 8 Discussion

TNBC is a major challenge in molecular and clinical oncology. Several targeted therapies for breast cancer have succeeded in the treatment of the luminal and HER2-positive subtypes, leading to notable improvements in both life expectancy and quality of life. However, TNBC remains immensely challenging due to the lack of specific therapeutic targets, the high mutational burden and tumor heterogeneity, the immunosuppressive tumor microenvironment, and the absence of biomarkers.

Over the past decade, advances in molecular understanding have led to the development of several targeted agents with clinically significant activity. A prime example is the use of PARP inhibitors in patients with germline BRCA1/2 or PALB2 mutations. However, the advent of immunotherapy with pembrolizumab and atezolizumab has been the most relevant achievement in TNBC in recent years (287, 292, 293). Unfortunately, the complexity of the mechanisms underlying the coexistence of the immune system and the tumor makes it challenging to generate more complex strategies.

Ongoing research in immunotherapy for triple-negative breast cancer (TNBC) is exploring various critical aspects. Non-cancerous cells in the tumor microenvironment (TME) play a pivotal role in tumor progression, including dysfunctional immune cells such as depleted T lymphocytes and immunosuppressive cells like MDSCs, TAMs, TANs, and Tregs. Additionally, stromal cells, such as CAFs, contribute to this environment by restricting the infiltration and effectiveness of antitumor T cells and dendritic cells, creating an imbalance between pro-tumor and antitumor cells.

Several signaling pathways, including Notch, IFNγ, and WNT, display alterations in the expression of their molecular components—such as ligands, receptors, and transcription factors—that facilitate tumor growth. Cancer cells exploit these modified expression patterns to support and accelerate their proliferation. Host immunity also influences the breast and gut microbiome, affecting tumor surveillance through shifts in signaling or metabolic processes tied to microbial activity. Notably, changes in the gut microbiome, driven by cancer, may either enhance or hinder response to immunotherapy.

Current studies are identifying numerous potential biomarkers and therapeutic targets, such as Jag-1, β-catenin, STAT3, and MUC1, while other immunosuppressive metabolites are under investigation as possible predictors of chemotherapy response. This multifaceted approach underscores the complexity and promise of advancing immunotherapeutic strategies for TNBC.

In conclusion, TNBC highlights the complexity of immunogenic mechanisms driving cancer progression. Despite extensive research into next-generation immunotherapies, current treatment options remain largely limited to the combination of immune checkpoint inhibitors with chemotherapy. Identifying new biomarkers of treatment sensitivity will help us better define the profiles of patients most likely to benefit from these emerging therapies, paving the way for more tailored and effective treatment strategies.
